# Biocontrol of Aflatoxigenic Maize Molds Using *Lactobacillus* spp.‐Based Formulations

**DOI:** 10.1002/fsn3.71039

**Published:** 2025-09-29

**Authors:** Inoussa Ilboudo, Hamidou Compaoré, Inoussa Compaoré, Sheryl Mounira Traoré, Laeticia Ella Dembélé, Fulbert Nikièma, Lanterbecq Déborah, Hagrétou Sawadogo‐Lingani, Elie Kabré

**Affiliations:** ^1^ Laboratory for the Study and Research of Soil Fertility and Production Systems (LERF) Nazi Boni University Bobo Dioulasso Burkina Faso; ^2^ National Agency for Environmental, Food, Occupational, and Health Product Safety (ANSSEAT) Ouagadougou Burkina Faso; ^3^ Department of Food Technology (DTA), Institute for Research in Applied Sciences and Technologies (IRSAT) National Center for Scientific and Technological Research (CNRST) Ouagadougou Burkina Faso; ^4^ Catholic University of West Africa (UCAO) Bobo Dioulasso Burkina Faso; ^5^ Laboratory of Biotechnology and Applied Biology Haute Ecole Provinciale de Hainaut‐Condorcet Mons Belgium

**Keywords:** aflatoxins B1, detoxification, *fura*, *gapal*, *Lactobacillus*, maize

## Abstract

Food contamination by mold is a serious and difficult problem to manage, leading to enormous economic losses. Mycotoxins have been recognized as one of the most dangerous contaminants in food. They can be toxic to humans and animals when they reach a certain ingestion threshold. These mycotoxins include aflatoxins, produced mainly by *Aspergillus* section *Flavi*, the most frequently isolated from food being *A. flavus* and *A. parasiticus* . Current control methods, mainly physical and chemical, are showing their limitations due to the environmental problems caused, resistance developed by pathogens, and the food organoleptic quality deterioration. Other control alternatives based on biocontrol can be explored. This present study aimed to isolate *Lactobacillus* spp. from local foods that can inhibit aflatoxigenic molds' growth and reduce aflatoxins production. To this end, four (04) strains (04) collection namely GNc, GNd, F3a, and G3Lab1 from *fura* and *gapal*, selected on the basis of their high antifungal potential, were co‐cultured with fungi reference strains (UBOCC‐A‐111042, UBOCC‐A‐106031, T1, and T2) and on maize aflatoxigenic molds (AF1, AF2, AF3, AP1, and AP2). Bioassays in vitro demonstrated that *Lactobacillus* spp. strains significantly inhibited fungal radial growth. Further tests were conducted on eleven (11) highly contaminated maize samples, showing a maximum reduction rate of 68% in the fungal flora. Analysis and quantification of residual aflatoxins B1, B2, G1, and G2 in maize grains using liquid chromatography coupled with tandem mass spectrometry (LC–MS/MS) revealed a decrease and, in some cases, complete suppression of aflatoxin B1, B2, G1, and G2 production in grains detoxified with *Lactobacillus* extracts.

## Graphical abstract

This study reviews *Lactobacillus* spp. use to combat aflatoxin contamination in food, specifically maize. Aflatoxins, produced by *Aspergillus* molds, are dangerous mycotoxins. Current control methods have limitations, prompting exploration of biocontrol alternatives. *Lactobacillus* strains isolated from local foods and co‐cultured them with aflatoxigenic mold strains. In vitro bioassays showed significant inhibition of fungal growth. Tests on contaminated maize samples demonstrated up to a 68% reduction in fungal flora. LC–MS/MS analysis revealed a decrease or complete suppression of aflatoxin production.

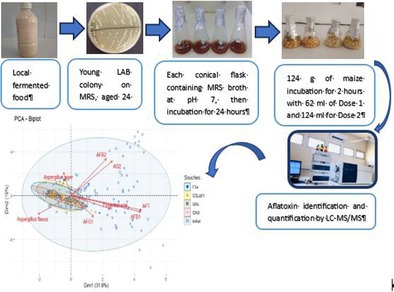

## 1 Introduction

Maize is the most widely cultivated cereal crop worldwide, whether consumed on our plates or for animal feed (Bretin‐Chabrol and Boehm 2024). Burkina Faso is a country where agriculture employs over 80% of the population. It also contributes significantly to the country's economy. However, the maize value chain development faces multiple obstacles, including grain quality due to varying production, harvesting, drying, and storage conditions, which hinder this sector (Somda et al. 2023). Maize production, in particular, is subject to aflatoxin contamination. These aflatoxins are caused by *Aspergillus*, section *Flavi* (Compaoré, et al. 2021). Molds and yeasts are the most common food spoilage organisms. These microorganisms are responsible for various food spoilage conditions. They cause poisoning due to the different mycotoxins secreted, which can over time harm the health of the consumer (Edah et al. 2021). This is not to mention the great economic losses throughout the world because spoiled food is food to be thrown away (Compaoré, Samandoulougou, Compaoré, et al. 2021; Pawlowska et al. 2012). Human beings have been looking for years for a way to fight against this scourge and to be able to get rid of it; despite the technological advancement and discoveries made in the field of science, it remains a great challenge for the food industries. Many physical and chemical methods have been developed in order to control fungal proliferation in food, and after many tests, it was concluded that the key to the mystery lies in biocontrol, and lactic acid bacteria prove to be an effective weapon in the fight against the growth of these fungi (Oranusi et al. 2013). Recently, many studies have focused on some microorganisms' use, including lactic acid bacteria (LAB), yeast, and fungi to remove mycotoxins from food (Assaf et al. 2018). Lactic acid bacteria are prokaryotic, heterotrophic, and chemo‐organotrophic cells. They are Gram‐positive, non‐spore‐forming, cocci or rods (Rodriguez 2023). They are generally immobile, lack catalase and oxidase, are facultative anaerobes, lack nitrate reductase, and have complex nutritional requirements for amino acids, peptides, vitamins, salts, fatty acids, and fermentable carbohydrates (Belketdid et al. 2022). They have the ability to extend the shelf life of food products by secreting numerous antifungal compounds; this is called biopreservation. The latter refers to the extension of shelf life and the improvement of food safety (Guan et al. 2010). The use of these bacterial species presents a very good alternative (Ouissem and Ikram 2020).

The preservation effect of lactic acid bacteria is linked to several factors such as organic acids and hydrogen peroxide formation, competition for nutrients, and antimicrobial substances production. The application of this method has been demanded by consumers since its inception, due to the desire to reduce the use of chemical preservatives due to their dangerousness (Ouissem and Ikram 2020).

With this in mind, we conducted this work to explore the ability of certain strains of bacteria of the genus *Lactobacillus* spp. from local fermented foods such as *gapal* and *fura* to control the development of pathogens potentially producing aflatoxins B1, B2, G1, and G2 on maize and to degrade aflatoxins already excreted in the cereal.

## 2 Materials and Methods

### 2.1 Sampling

The aim of our study was to isolate the *Lactobacillu*s present in local fermented foods and to develop formulations that can inhibit aflatoxigenic molds' growth and reduce aflatoxin production. To this end, nine (09) samples of *fura* and *gapal* were collected from local markets. Transport and storage conditions at room temperature were adopted to accelerate the fermentation process. Four (04) strains from these samples were selected on the basis of their high antifungal potential on fungal strains. These lactic strains were tested on eleven (11) samples of maize highly contaminated with aflatoxins.

### 2.2 Microorganisms

The strains used for the antifungal tests belong to a single group of microorganisms belonging to the *Aspergillus* section *Flavi*. These are *Aspergillus flavus* UBOCC‐A‐106031 and *Aspergillus parasiticus* UBOCC‐A‐111042 (Table 1). These two strains are aflatoxin‐producing reference strains provided by the Food Technology Department (DTA) of IRSAT. Two *Aspergillus flavus* T1 and T2 isolated from maize were also used as reference strains.

**TABLE 1:** *Aspergillus* section *Flavi* reference strains used in the study.

Strains	References	Origin of sustrate	Country of origin	Source
*Aspergillus parasiticus* var. *globasus*	UBOCC‐A‐111042	Unknown	Japan	UBO Japan
*Aspergillus flavus*	UBOCC‐A‐106031 T1	Maize Maize	France et Burkina Faso	UBO France Compaore et al. (2016)
	T2	Maize	Burkina Faso	Compaore et al. (2016)

Both were also identified by molecular biology in previous work (Compaore et al. 2016). The strains were first revived on Sabouraud medium before being preserved in PDA medium at 4°C throughout the study.

*Aspergillus* section *Flavi* strains were isolated from maize samples, using the Ulster method or the direct method (Ouattara‐Sourabie et al. 2011). It consisted deposing the maize grains directly onto the Sabouraud isolation medium enriched with chloramphenicol at a rate of ten (10) seeds per dish after wetting the seeds. To do this, about 60 seeds from each sample, taken at random, were introduced into a petri dish containing three (3) layers of blotting papers previously soaked in sterile distilled water and then placed in a radiation room on shelves for 1 week at 37°C. Then a procedure for isolating the interest strains was carried out on the Potato Dextrose Agar (PDA) medium by subculturing using a loop followed by incubation at 25°C for 72 h. Growing molds were visually observed on the agar surface based on their color and shape. Molds with green, white, and yellow color were randomly sampled.

Furthermore, the *Lactobacillus* spp. used in this work were isolated on Man Rogosa Sharpe (MRS) medium according to the method described by Di Cagno et al. (2011) For each sample (*gapal* and *fura*), a total of 50 g was suspended in 50 mL of sterile MRS broth (Liofilchem, Italy) used as enrichment medium and incubated at 37°C for 72 h. After enrichment, decimal dilutions (10^−3^ to 10^−6^) were performed according to ISO 6887‐1 (2017). Inoculation was done in the mass with MRS agar (Liofilchem, Italy). The inoculated petri dishes were placed in the anaerobic jars (Biolab) with anaerocult CO_2_ generators (Merck KGaA, Germany). The jars were hermetically sealed and incubated in an incubator (Binder) at 37°C for 48 h.

The dishes containing 15 to 150 isolated colonies were then selected by isolation. On each of the selected dishes, ten (10) characteristic and distinct colonies were isolated and purified by sequential inoculation of a single colony in MRS broth, followed by streaking on MRS agar until pure colonies were obtained. The purity of the colonies was verified by visual observation of the morphology, size, and appearance of the colonies and by microscopic observation. The purified isolates were introduced into MRS broth cryotubes (Liofilchem, Italy) with 30% glycerol (87%) and stored in a freezer at −20°C. A total of four (04) strains were retained; these are the G3Lab 1, GNc, and GNd strains from *gapal* and the F3a strain from *fura*.

### 2.3 In Vitro Antagonism Test

*Lactobacillus* spp. isolates were tested for their antifungal activity against *Aspergillus flavus* T1, T2, and UBOCC‐A‐106031 and *Aspergillus parasiticus* var. *globasus* UBOCC‐A‐111042 reference strains for primary screening. For secondary screening, *Aspergillus* section *Flavi* isolated from maize in this study was used. Antifungal activity was determined using the overlay technique or double‐layer agar (Schnürer and Magnusson 2005). This method consisted of four steps: pure culture of *Lactobacillus* spp., preparation of a monospore suspension, detection of antagonistic lactic acid bacteria using the overlay technique, and reading the antifungal assay. Lactic acid bacteria isolates were first streaked in 2 cm lines on MRS, then incubated at 30°C for 48 h to allow anaerobic growth. The colonies obtained were then covered with 10 mL of sweet malt extract (0.7% agar) containing 0.1 mL of monospore suspension (10^3^ spores/ml) and incubated at 30°C. After 48 h or 72 h of incubation, the inhibition zones were evaluated around the bacterial streaks according to the following criteria: (−): absence of inhibition zone; (+): inhibition zone between 0.1% and 3% of the surface of the Petrie dish; (++): inhibition zone between 3% and 8% of the surface of the Petrie dish; (+++) inhibition zone greater than 8% of the surface of the Petrie dish (Figure 4). Inhibition assays were performed in duplicate. Isolates that showed the highest activity (++ and +++) were selected for inhibitory activity on fungal spore germination.

### 2.4 In Vivo Antagonism Test on Collected Maize Kernels

Bacterial broths based on pure LAB isolates were used for maize kernel decontamination tests. The culture broths were administered in two doses. For dose 1 (D1), 124 g of maize was soaked in 62 mL, while for dose 2 (D2), the amount was increased to 124 mL.

**FIGURE 1:**
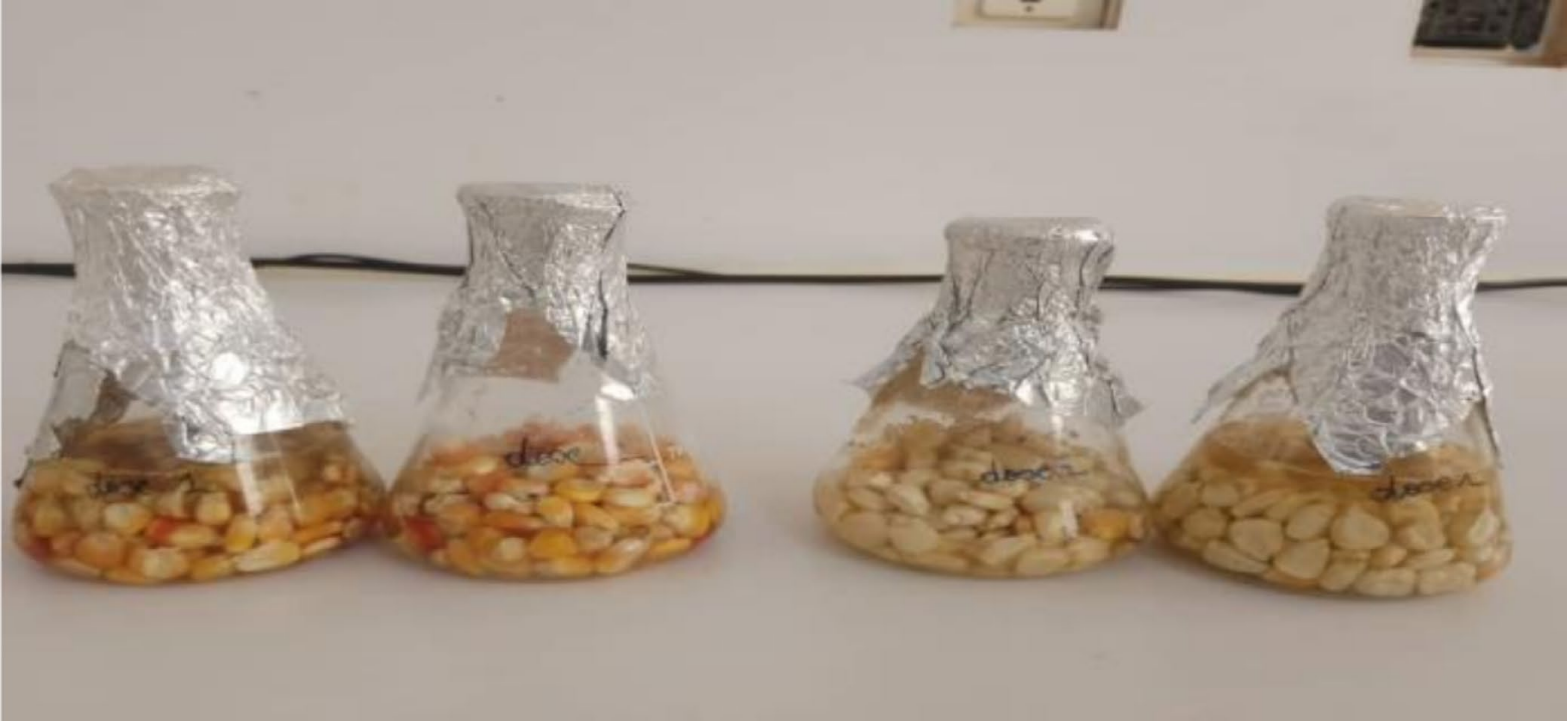
Samples incubated in *Lactobacillus* broths.

For each treatment, the maize kernels were weighed and placed in airtight glass jars before adding the appropriate amount of bacterial broth according to the dose. The jars were then capped, and the contents were thoroughly mixed. Soaking lasted 3 h; then the kernels were drained and air‐dried under a fume hood for 36 h. A control composed of untreated maize served as a reference base for results evaluation (Figure 1).

**FIGURE 2:**
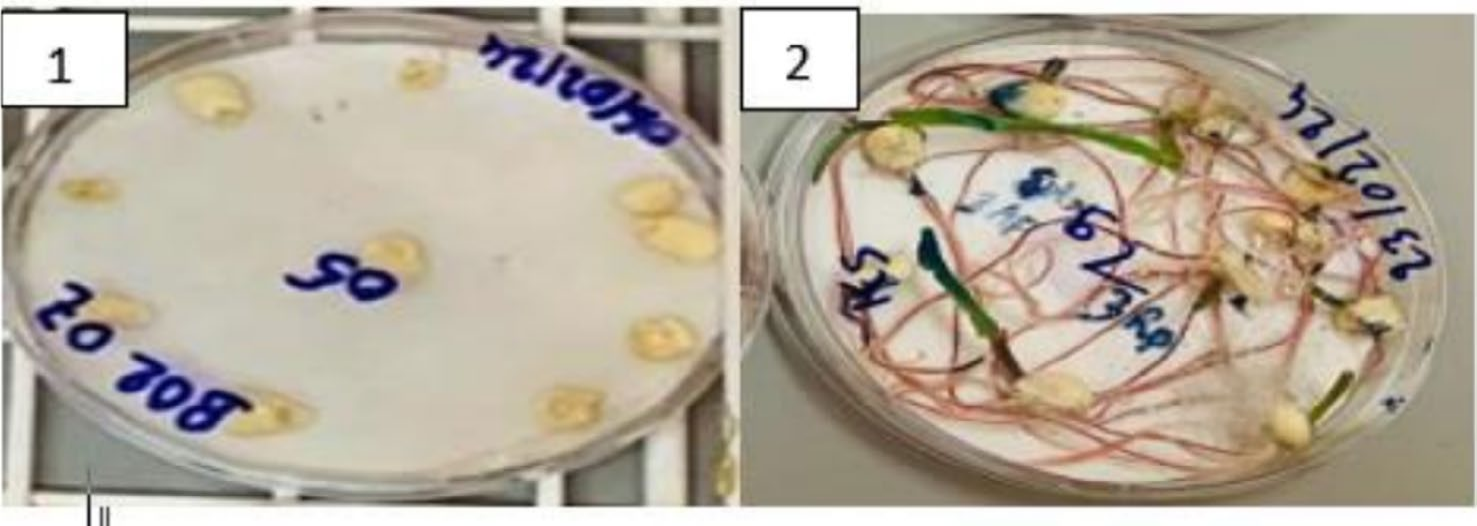
Seeds placed on blotting paper in a Petri dish before incubation (1); mold development on the seeds after 7 days of incubation at 25°C (2).

The standard blotting paper method, described by Mathur and Kongsdal (2003), was used with slight modifications to detect a wide range of molds capable of growing on seeds in the presence of moisture. All collected samples (60 samples) were subjected to sanitary analysis. Thus, two hundred (200) seeds from each sample (treated and untreated) were randomly selected and placed in Petri dishes (90 mm in diameter) containing three layers of moistened blotting paper, with 10 seeds per dish (Figure 2).

The dishes were placed in an incubation chamber at 25°C, under alternating light cycles of 12 h of near‐UV light and 12 h of darkness per day, for 7 days. Each sample was analyzed using a randomized Fisher block design with 4 replicates, that is, 50 seeds per replicate. A preliminary identification of each fungal species present on the seeds was made by direct observation under a stereomicroscope followed by examination of the mycelium and/or conidia under a microscope, referring to the mold identification manual by Mathur and Kongsdal (2003). Maize *Aspergillus* section *Flavi* isolate identification was also carried out by comparing their morphological and biochemical characteristics with those of reference fungal strains. The fungal species present on each seed were recorded, along with the number of infected seeds.

The maize kernel infestation rate was determined by counting infected maize kernels relative to the total kernels per sample.

### 2.5 Aflatoxins B1, B2, G1, and G2 Determination in Maize

Maize samples in which aflatoxin‐producing strains were identified were analyzed by LC–MS/MS (liquid chromatography–tandem mass spectrometry) to quantify their aflatoxin content.

The principle involved extracting aflatoxin from maize grain samples using suitable organic solvents, followed by purifying this aflatoxin on an immunoaffinity column before identifying and quantifying it. Thus for each sample 25 g were ground in which 125 mL of methanol/water (70/30), the whole was stirred for 20 min then filtered and 15 mL of the filtrate was made up to 45 mL before taking 15 mL for purification with IAC columns (Libios) then injected into the LC–MS/MS for detection and quantification.

#### 2.5.1 LC–MS/MS Chromatographic Analysis

The filtrate obtained after extraction of the various maize grain samples was placed in vials for subsequent analysis. The Agilent Technology 1290 Series coupled with the 6430 mass spectrometer used for the separation is composed of the following modules: (i) Injection volume set at 10 μL. (ii) A constant flow rate of 0.4 mL/min in isocratic mode. (iii) Chromatographic separation was performed using a Zorbax Eclipse XDB C18 column (50 × 4.6 mm, 1.8 μm) (Agilent, USA). (iv) Mobile phase A: Water/formic acid (99/1 v/v) and 10 mM ammonium formate. (v) Mobile phase B: methanol/water/formic acid (97/2/1 v/v/v) and 10 mM ammonium formate. The column temperature was maintained at 40°C. Standards of AFB1, AFB2, AFG1, and AFG2 (purity ≥ 99%) were purchased from Sigma‐Aldrich Chemical Co. (St. Louis, MO, USA).

#### 2.5.2 Quality Assurance and Quality Control

The analytical method has been validated in accordance with ISO 16050:2003. Bipea Laboratory (France) provided the quality control material. For AFB1, AFB2, AFG1, and AFG2, the regression equations and correlation coefficients were: *y* = 0.9955*x* + 0.0979 (*R* ^2^ = 0.9994), *y* = 1.0083*x* − 0.1789 (*R* ^2^ = 0.9996), *y* = 1.001*x* − 0.0225 (*R* ^2^ = 0.9991), and *y* = 1.0096*x* − 0.2076 (*R* ^2^ = 0.9997), respectively. All correlation values (*R* ^2^) reached 0.999, indicating good linearity. The recovery rates obtained were 87% for AFB1, 95% for AFB2, 105% for AFG1, and 102% for AFG2.

### 2.6 Statistical Analyses

Aflatoxin database levels obtained before and after treatment and by replicates using the LC–MS/MS method were created using Excel version 2021 and converted into csv format. This database was then imported into RStudio software (2024.12.0). Data were subjected to the analyses of variance (ANOVA), and significant differences between means were revealed via the Tukey test (*p* < 0.05), which was done using XLSTAT (2016) software for statistical analyses. Principal component analysis (PCA) was performed using RStudio software.

## 3 Results and Discussion

### 3.1 In Vitro Antagonism Test

#### 3.1.1 *Lactobacillus* spp. Characteristics

Eleven (11) *Lactobacillus* spp. were isolated and purified from *gapal* and *fura* samples. Of these 11 isolates, four (GNc, GNd, G3Lab1, and F3a) were selected on the basis of their pronounced antifungal activity, and they will be used for in vivo antagonism tests. These isolates were characterized after purification based on visualization with the naked eye. The morphological and biochemical characteristics of these isolates were Gram‐positive, catalase‐negative, and oxidase‐negative.

**FIGURE 3:**
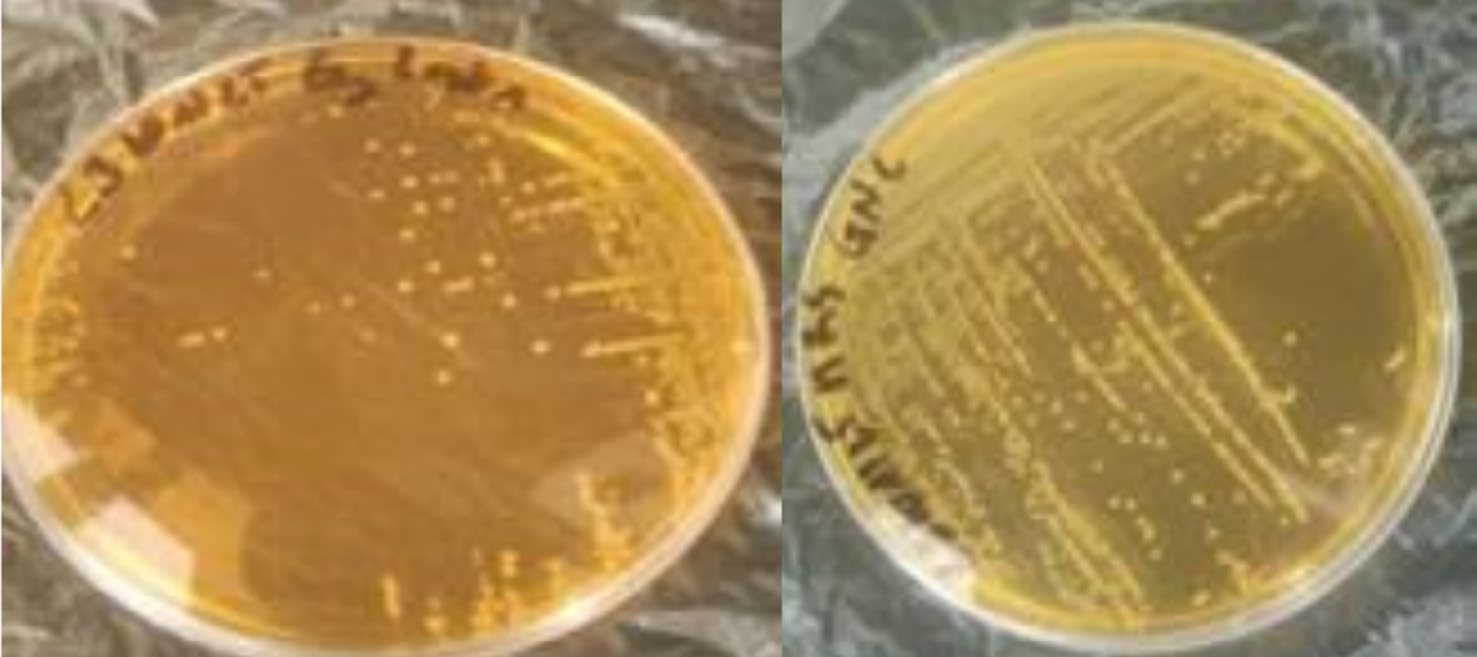
Young *Lactobacillus* spp. colonies aged 24 h.

Isolates observation on MRS agar revealed, after purification, round or lenticular colonies with a homogeneous outline, white or gray in color, which were not attached to the agar (Figure 3).

#### 3.1.2 Microscopic Aspects

**FIGURE 4:**
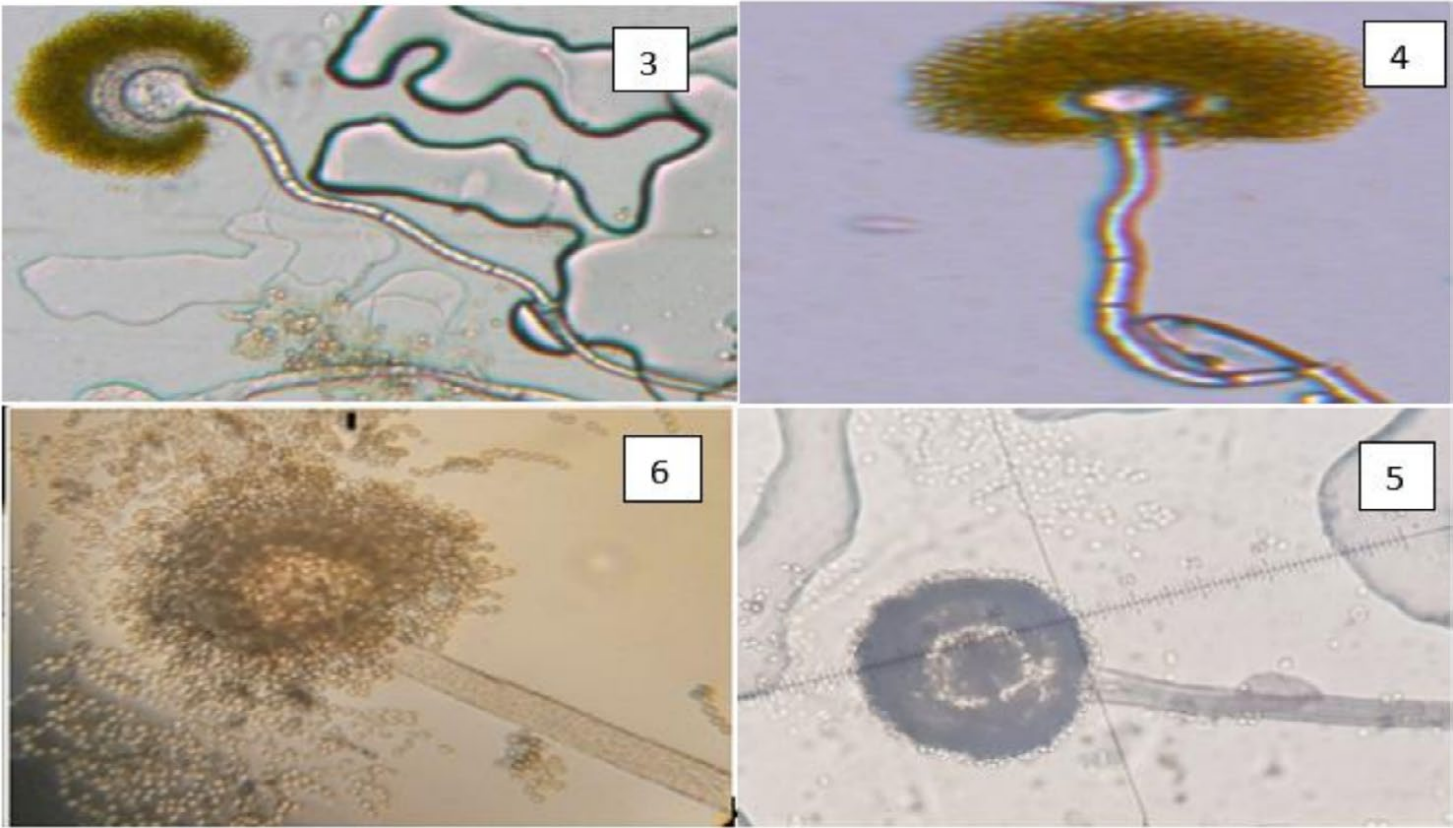
Microscopic aspects of *Aspergillus* isolates from the *Flavi* section (G × 100) of the *Aspergillus* heads of strains AF1 (3), AF2 (4), AP1 (5), and UBOCC‐A‐106031 (6).

Figure 4 shows microscopic observations results (G × 100) of the reference strains *Aspergillus flavus* T1, T2, UBOCC‐A‐106031 and *Aspergillus parasiticus* UBOCC‐A‐111042, as well as the local strains. Figure 4 (3–6) shows AF1, AF2, AP1 and UBOCC‐A‐106031 head, respectively (Table 2).

**TABLE 2:** Strains studied microscopic characteristics.

Souches	Code	Texture du pied	Série	Longueur conidiophore (μm)	Diamètre des vésicules (μm)	Taille des conidies (μm)	Tête/forme de conidies
Souches locales	AF1	Lisse	Bisériée	400–950	40–45	2–7	Rayonnante
	AF2	Rugueux	Bisériée	200–500	15–40	3–5	Colonne courte
	AF3	Lisse	Unisériée	300–550	30–34	3–5	Rayonnante
	AP1	Lisse	Unisériée	200–700	15–20	2–7	Rayonnante
	AP2	Lisse	Unisériée	200–500	25–45	4–6	Rayonnante
Souches de références	T_1_	Rugueux	Bisériée	200–450	20–45	3–5	Rayonnante
	T_2_	Rugueux	Bisériée	400–800	14–43	3–5	Rayonnante
	UBOCC‐A‐106031	Rugueux	Bisériée	450–930	14–43	3–5	Rayonnante
	UBOCC‐A‐111042	Lisse	Unisériée	200–430	28–32	4–7	Rayonnante

The hyaline, non‐partitioned conidiophore is visible, as is the spheroidal, globular vesicle.

#### 3.1.3 Antifungal Activity of *Lactobacillus* spp.

**FIGURE 5:**
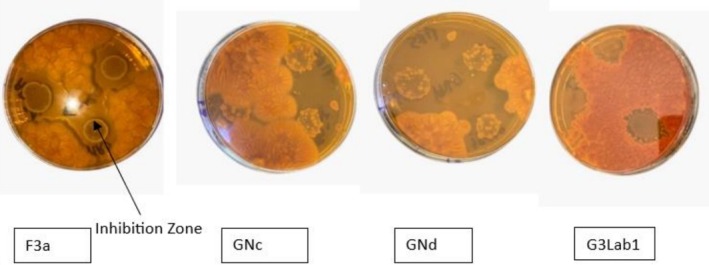
Antifungal effects of four *Lactobacillus* spp. isolates were observed: F3a exhibited activity against AF2, GNc was effective against AP1, GNd showed activity towards strain AF2, and G3Lab1 against AF ATTC.

**FIGURE 6:**
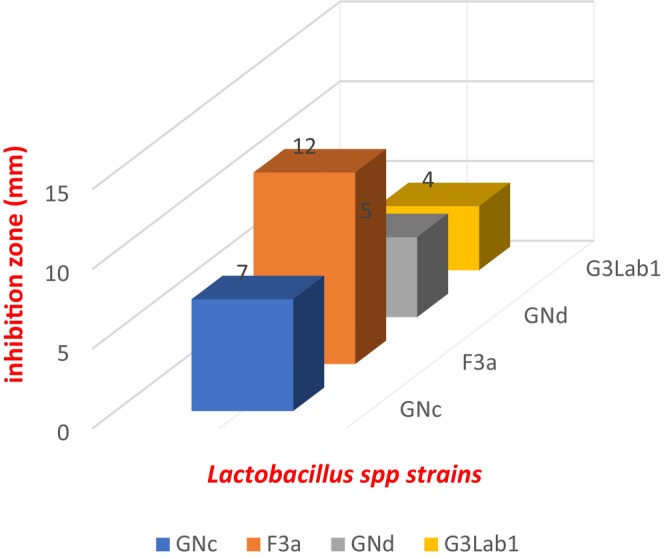
Inhibition zone in mm depending on the strains used.

In vitro antagonism test results showed that testing the four strains revealed fungal growth inhibition. This inhibition varies depending on the bacterial strain and target mold strain for testing (Figures 5 and 6).

These results can be explained by the fact that the nutritional components of each strain play an important role in the antifungal metabolites production by *Lactobacillus* (Assaf et al. 2019; Li et al. 2024). The difference in growth inhibitory effect of mycelium may be due to the growth strain; thus, the strain used can influence the degree of antibiosis manifested by the antagonists in question (Gargouri‐kammoun et al. 2016).

### 3.2 In Vivo Antagonism Test on Collected Maize Kernels

**FIGURE 7:**
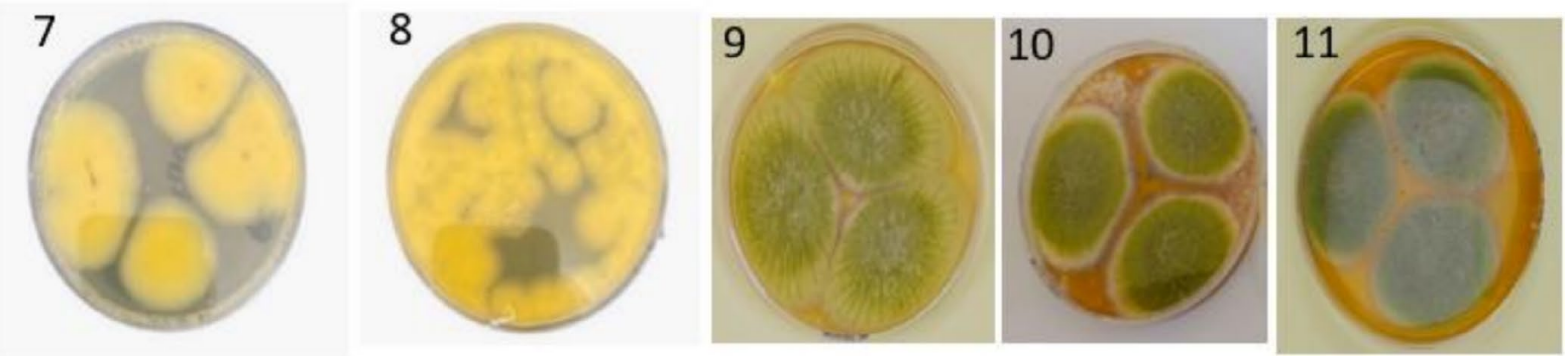
Visible morphological characteristics of *Aspergillus flavus* strains (7–9) and *Aspergillus parasiticus* (10, 11) grown on PDA after a week of incubation at 30°C.).

Maize samples were used as a matrix for mold isolation. From there, a naked‐eye observation of the consortia (Figure 7) was made to retain only molds forming white, yellow, and green colonies. A total of five (5) isolates were retained for the morphological characterization of *Aspergillus* strains in comparison with the reference fungal strains.

Figure 7 below shows the macroscopic characteristics on the 5th day of incubation.

The effectiveness of the different treatments in reducing the mold rate produced by contaminated grains was evaluated. The treatments included the application of lactic acid strains F3a, GNd, GNc, and G3Lab1.

**FIGURE 8:**
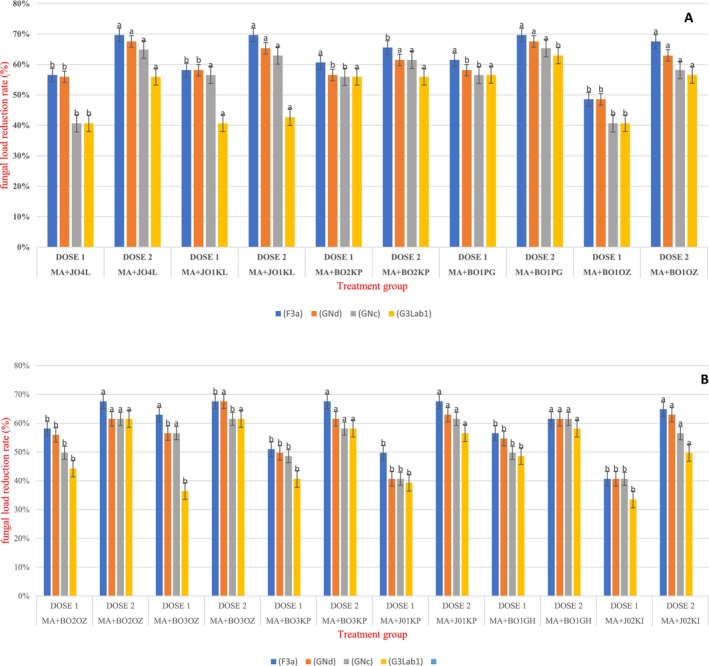
Treatment efficacy on molds associated with five (A) and six (B) highly contaminated maize samples. The different letters are from the Turkey test performed. They show the difference between the reduction rates depending on the doses used.

The treatments showed variations in their effectiveness depending on the lactic acid type and the dose administered. This was the case for the F3a strain, which significantly reduced *Aspergillus* growth by approximately 68% for all maize samples decontaminated at dose 2% and 58% for dose 1 (Figure 8).

**FIGURE 9:**
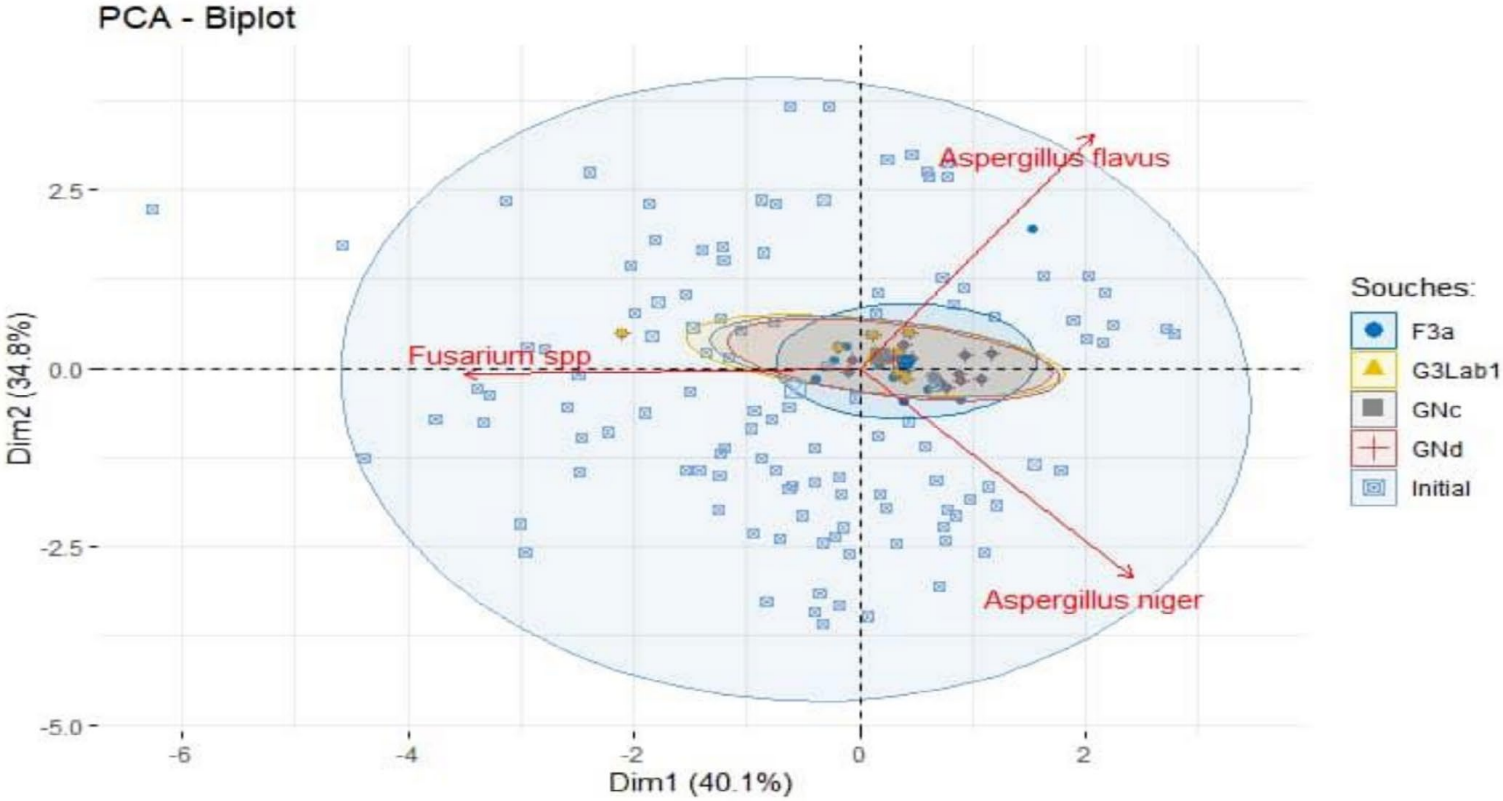
PCA analysis of various molds observed in maize cultivars based on the different treatments applied.

The PCA of the different molds counted in maize cultivars according to the different treatments was illustrated in Figure 9 with a total representativeness of 74.9%, including 40.1% on axis 1% and 34.8% on axis 2. Indeed, *Aspergillus flavus* and *niger* contribute, respectively, to the formation of axis 2, while *Fusarium* contributes to axis 1. This biplot shows that maize treated with the bacterial strains resulted in a good reduction in mold.

### 3.3 Aflatoxins B1, B2, G1, and G2 Determination in Maize

**FIGURE 10:**
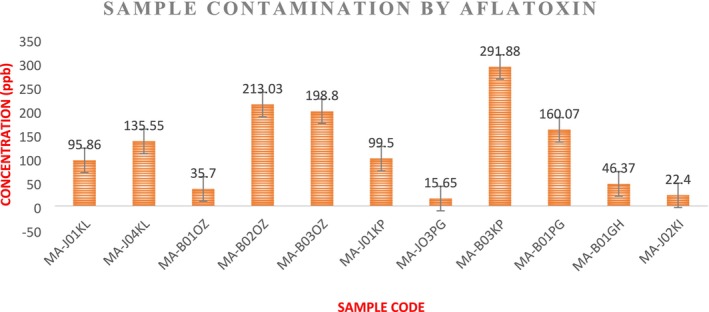
Results of aflatoxin assay at initial (T0) in maize samples (ppb).

The total aflatoxin determination results at T0 showed high contamination of maize samples by aflatoxins, particularly aflatoxin B1. The values ranged from 15.65 to 291.88 ppb obtained in samples MA‐JO1KL, MA‐JO4KL, MA‐BO1OZ, MA‐BO2OZ, MA‐BO3OZ, MA‐JO1KP, MA‐JO3PG, MA‐BO1PG, MA‐BO1GH, and MA‐JO2KI, as shown in Figure 10. A total of 87.09% of the samples were contaminated with aflatoxin B1. Eleven (11) main samples had the highest total aflatoxin concentrations.

**FIGURE 11:**
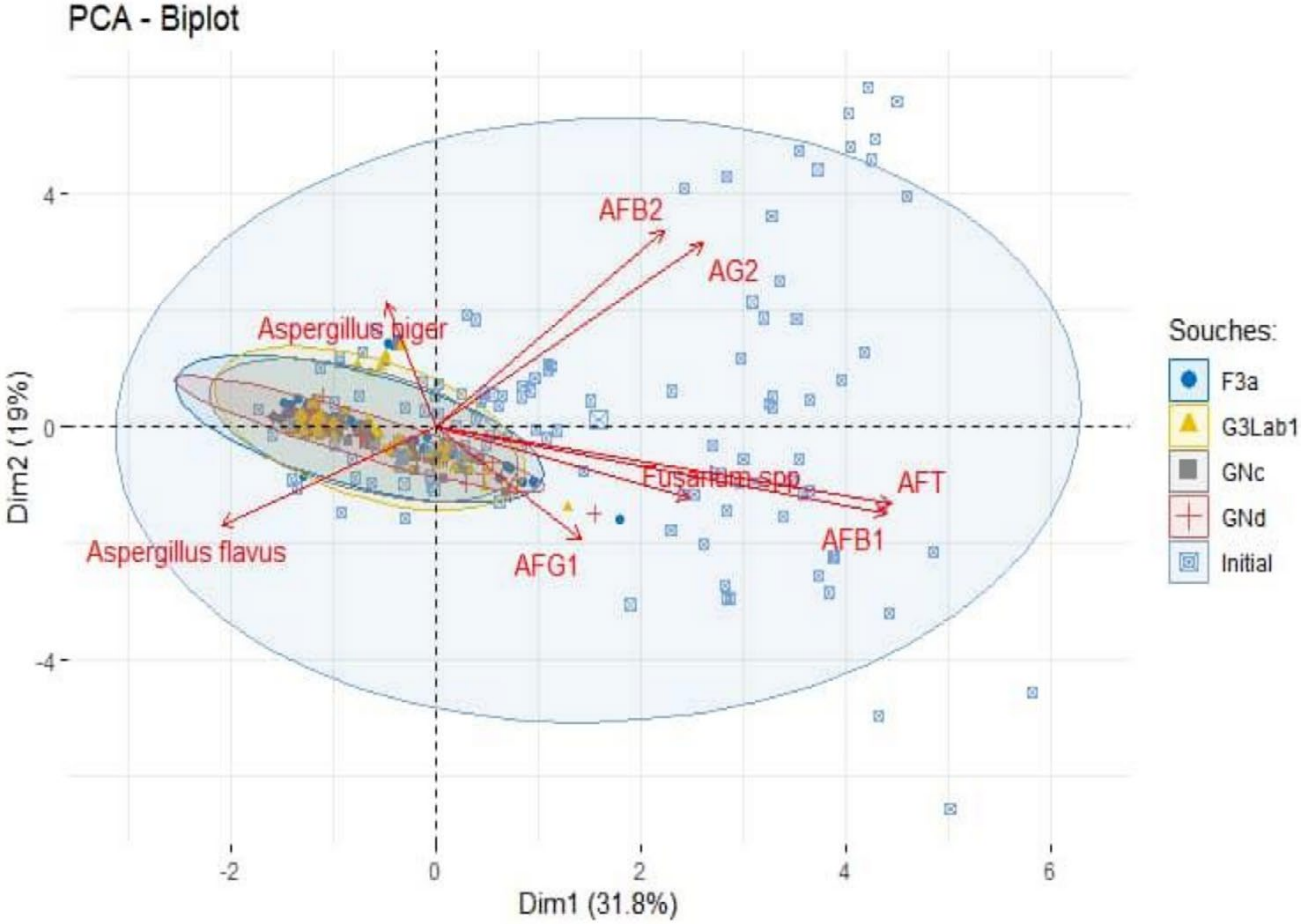
PCA analysis of aflatoxin levels in various maize cultivars based on different treatment methods.

The PCA of the aflatoxin content of different maize cultivars according to the different treatments was illustrated in Figure 11 with a total representativeness of 70.4%, including 43.2% on axis 1% and 27.2% on axis 2. This biplot shows a significant difference at the 5% level between the maize samples at baseline and those treated with bacterial strains. This would mean that the strains significantly reduced aflatoxin content. Among the biplots, the smallest showed a significant reduction. These biplots are represented in order of reduction by GNd, GNc, G3Lab1, and F3a.

After treating the most aflatoxin‐contaminated samples with formulations based on lactic acid F3a, GNc, GNd, and G3Lab1 at different doses (D1: 62 mL; D2: 124 mL), significant reduction rates were obtained, especially for aflatoxin B1, which was the most prevalent (Table 3). The percentage reduction was calculated using the following formula:

$$R\left(\%\right)=\frac{A-B}{A}\times 100$$

**TABLE 3:** Aflatoxin reduction (*R*) rate depending on the dose and strain used for detoxification.

Code	Strains															
	G3Lab1				GNd				F3a				GNc			
	*R* rate D1	*R* rate D2	*p*	Signif.	*R* rate D1	*R* rate D2	*p*	Signif.	*R* rate D1	*R* rate D2	*p*	Signif.	*R* rate D1	*R* rate D2	*p*	Signif.
MA‐J01KL	7.2 ± 0.5^a^	12.1 ± 4.9^b^	0.002	**	6.63 ± 0.11^a^	8.13 ± 0.06^b^	< 0.001	***	26.67 ± 0.31^a^	28.93 ± 1.15^b^	0.018	*	14.43 ± 10.52^a^	14.93 ± 9.90^a^	0.937	ns
MA‐J04KL	60.1 ± 0.8^c^	65.4 ± 1.4^d^	< 0.001	***	67.86 ± 0.15^c^	68.89 ± 0.57^d^	0.002	**	66.53 ± 0.12^c^	67.27 ± 0.39^d^	0.007	**	78.40 ± 0.45^b^	81.43 ± 0.76^c^	< 0.001	***
MA‐B01OZ	42.6 ± 0.4^e^	45.6 ± 3.2^f^	0.021	*	40.71 ± 0.16^e^	44.45 ± 0.42^f^	< 0.001	***	34.67 ± 1.65^e^	41.63 ± 0.39^f^	< 0.001	***	39.07 ± 1.00^d^	43.97 ± 2.15^e^	0.003	**
MA‐B02OZ	71.0 ± 0.2^g^	71.9 ± 0.6^g^	0.041	*	67.19 ± 0.05^g^	67.50 ± 0.32^g^	0.121	ns	67.27 ± 0.06^g^	67.90 ± 0.42^g^	0.078	ns	68.47 ± 0.35^f^	70.37 ± 0.95^g^	0.006	**
MA‐B03OZ	99.5 ± 0.1^h^	99.7 ± 0.2^h^	0.150	ns	99.39 ± 0.01^h^	99.69 ± 0.01^i^	< 0.001	***	99.80 ± 0.00^h^	100.00 ± 0.00^h^	0.083	ns	99.10 ± 0.10^h^	99.80 ± 0.00^i^	< 0.001	***
MA‐J01KP	87.7 ± 0.6^i^	89.6 ± 0.8^j^	0.002	**	85.26 ± 0.02^j^	86.66 ± 0.06^k^	< 0.001	***	83.60 ± 0.25^i^	84.20 ± 0.30^j^	0.010	*	85.70 ± 0.30^j^	88.20 ± 1.15^k^	0.011	*
MA‐JO3PG	36.5 ± 1.2^k^	39.1 ± 4.5^l^	0.047	*	35.72 ± 0.45^l^	36.91 ± 0.07^m^	0.012	*	31.50 ± 0.45^k^	35.47 ± 1.25^l^	0.003	**	22.53 ± 1.05^l^	26.30 ± 4.15^m^	0.082	ns
MA‐B03KP	64.5 ± 0.4^m^	65.7 ± 0.5^n^	0.018	*	58.55 ± 0.01^n^	59.53 ± 0.02^o^	< 0.001	***	54.20 ± 1.20^m^	56.13 ± 0.36^n^	0.039	*	62.33 ± 0.35^n^	64.30 ± 0.90^o^	0.004	**
MA‐B01PG	92.1 ± 0.4 ^o^	93.5 ± 0.9^p^	0.021	*	92.42 ± 0.07^p^	92.46 ± 0.03^p^	0.645	ns	91.30 ± 0.10^o^	93.10 ± 0.10^p^	< 0.001	***	92.50 ± 0.10^p^	94.07 ± 0.58^q^	< 0.001	***
MA‐B01GH	96.5 ± 0.7^q^	97.6 ± 0.7^r^	0.032	*	93.45 ± 0.04^q^	93.65 ± 0.12^r^	0.043	*	94.60 ± 0.20^q^	96.87 ± 1.35^r^	0.024	*	93.70 ± 0.40^r^	98.83 ± 2.05^s^	0.004	**
MA‐J02KI	90.8 ± 1.2^s^	93.1 ± 1.6^t^	0.008	**	91.82 ± 0.51^s^	91.65 ± 0.07^s^	0.587	ns	87.53 ± 0.31^s^	88.87 ± 1.15^t^	0.048	*	78.03 ± 1.65^t^	89.90 ± 3.45^u^	0.001	**

*Note:* Signif.: ***p < 0.001, **p < 0.01, *p < 0.05, ns = *p* > 0.05. The superscript letters (a‐u) are used to indicate the relationships between the different doses administered and the reduction rates with a *p*‐value of 5%.

with A: The amount of total aflatoxin in the untreated maize sample and B: The amount of total residual aflatoxin in the detoxified sample.

**FIGURE 12:**
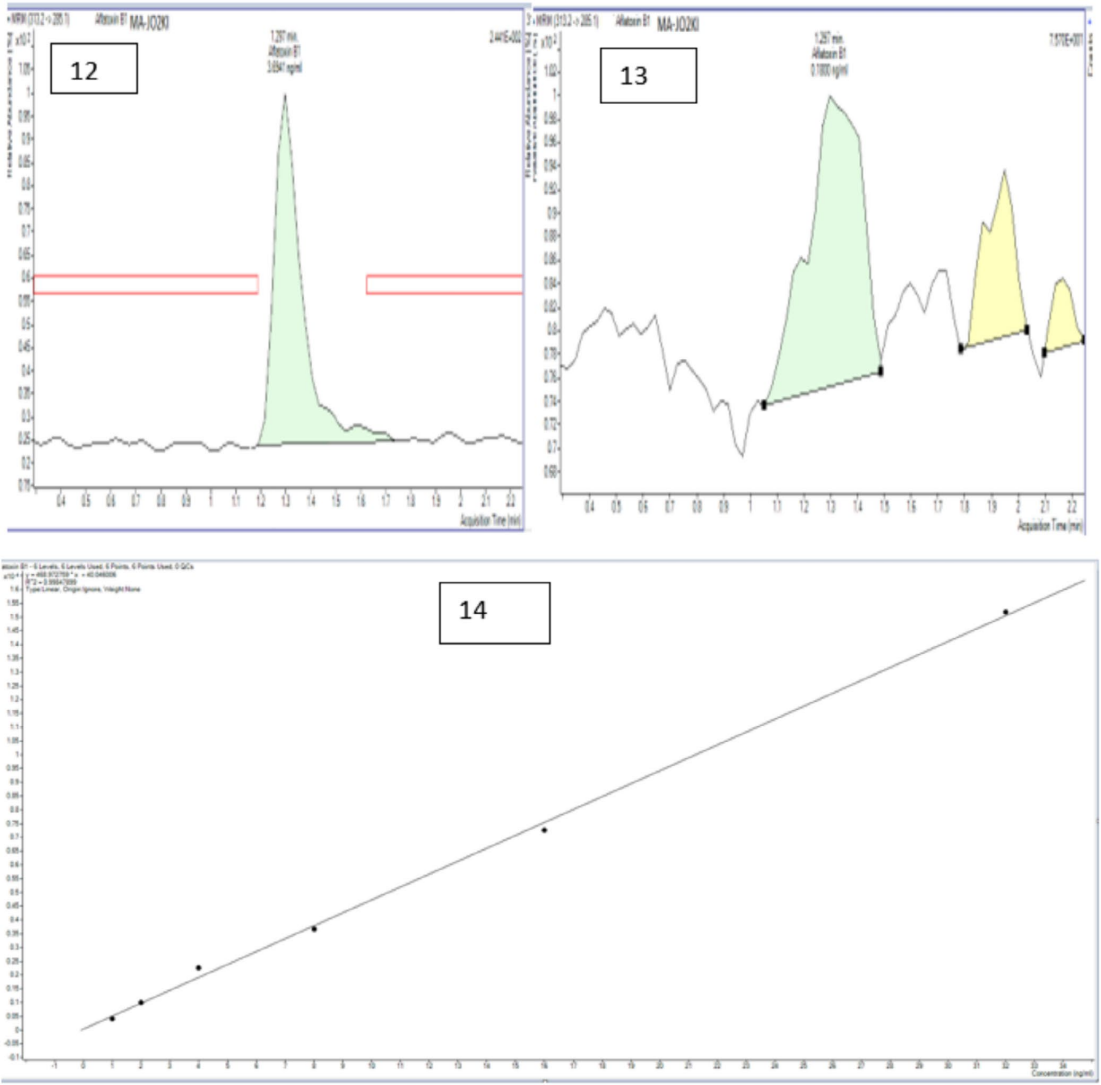
Treatment impact on toxin levels: (12) sample MA‐JO2KI chromatogram showing 22.4 ppb prior to treatment, (13) sample MA‐JO2KI chromatogram revealing 5.34 ppb post‐treatment with the F3a strain, (14) calibration curve for aflatoxin B1.

The four (04) lactic acid bacteria‐based formulations showed reduction rates ranging from 6.63% to 100%. Averaging the reduction rates of the different lactic acid bacteria‐based formulations with the two doses D1 and D2, almost equal effectiveness was observed, with reduction rates ranging from 67.5% to 68.4%. The F3a‐based formulation achieved the highest rate (68.4%) (Figure 12).

The biological detoxification of mycotoxins is defined as the usage of microorganisms, as well as their microbial enzymes and metabolites for mycotoxin binding and potential degradation (Muhialdin et al. 2020). The microorganisms implicated in biological degradation should follow certain standards such as being safe, nonpathogenic, possessing mycotoxin degrading ability, pertaining activity during packing, not forming improper odors or taste, and preserving the nutrient value of food (Varga and Tóth 2005). One of the essential conditions for a microorganism to be considered a possible probiotic is that it can inhibit the growth of the target pathogen, demonstrating an antagonistic action by inhibiting it when both are in the same environment. To date, there are two theories by which LAB eliminates toxins: one through physical adsorption and the other through biodegradation of mycotoxins. Researchers have conducted several detoxification experiments to test which theory is more adequate. Experiments with thermally inactivated bacteria provoked higher detoxification of mycotoxins as compared to activated cells. Studies showed that the binding of mycotoxins by microorganisms is a rapid process, which forms a reversible complex between the toxin and the bacterial surface without altering the mycotoxins' structure (Bueno et al. 2007). Shetty and Jespersen's investigations revealed that the detoxification process is related to a physical union between the mycotoxins and the bacterial cell components, instead of covalent binding or biodegradation by bacterial metabolism (Shetty and Jespersen 2006). Yiannikouris et al. (2006) reported that hydrogen bonds and Van der Waals interactions may be implicated in this binding mechanism. On the other hand, according to Hernandez‐Mendoza et al. (2009), the differences in the mycotoxins' binding ability of various *Lactobacillus* strains could be explained by the differences in the cell wall components, specifically teichoic acid and peptidoglycan contents. Different structures in the cell wall of microorganisms are responsible for the mycotoxin binding capacity. Cell walls comprise carbohydrates (peptidoglycan, mannose, and glucan), proteins, and lipids, which may offer different binding sites (Wang et al. 2022). However, there are arguments among different researches on the specific cell wall components implicated in the binding processes, such as glucogalactans and β‐glucans (Taheur et al. 2017), mannoproteins (Caridi et al. 2012), and β‐glucans and mannans (Pereyra et al. 2015). Therefore, in the interaction of bacterial cells and mycotoxins, it appears that various binding mechanisms may be implicated involving non‐covalent bonding, hydrophobic interactions, ionic interactions, or hydrogen bonds (Huwig et al. 2001; Ringot et al. 2007). The benefits of the biological detoxification process include its easiness, cost‐effectiveness, applicability over a broad range of target mycotoxins, efficacy in a wide range of fluid and foodstuffs, and its insignificant effects against nutrients naturally found in food (Varga and Tóth 2005).

Subsequent work by Khan et al. (2004) showed that the content of maize kernels was rarely reduced by biological treatments even though the severity of the disease was significantly reduced by the species: *Bacillus subtilis* AS433, *B. subtilis* AS434, *B. subtilis* OH131.1. These data are in contrast to the results found in this study, which showed that *Lactobacillus* bacteria significantly reduced the level of total aflatoxins in maize kernels. Moreover, the results found are in agreement, which showed that the antagonism exerted by the presence of specific bacteria (*Bacillus* spp.) can influence the production of mycotoxins.

Mycotoxins binding in several studies was reported to be rapid, and the binding percentages were generally affected by many factors such as incubation time, type of bacteria, bacterial concentration, pH, type of medium, and temperature (Ringot et al. 2007). The use of bacterial adsorbents in food presents several advantages over chemical and physical detoxification methods. The bacterial detoxification assay is considered more effective and highly specific, especially since the binding affinity of mycotoxins varies not only among different species but also among different strains within the same species.

The treated samples that showed the highest reduction rates were those decontaminated by F3a. These reduction rates are lower than those obtained by Traoré (2022) on maize decontaminated with liquid formulations based on lactic acid bacteria, which were between 60.36% and 100%. This could be explained by the impact of certain physical parameters such as pH, humidity level, and aflatoxin content at T0, which was higher in the present study, and the temperature of the environment. Indeed, according to Dushimeyesu et al. (2021), Jaffar et al. (2023), and Leghouini (2022) the differences between the reduction rates could be attributed to some factors that affect the biodegradation mechanism of aflatoxin, such as the pH and incubation time of the isolates, the nature of the sample, and even the samples' contamination level. This hypothesis is confirmed by the work carried out by Ramos et al. (2022) and Tremonte et al. (2017), which demonstrated that there was a relationship between environmental conditions and the antimicrobial activity of *Lactobacillus Plantarum* .

According to Laref et al. (2013); Price‐Christenson and Yannarell (2023) the antiaflatoxigenic activity of lactic acid bacteria would be due to the bacteriocins and organic acids they produce. Indeed, the inhibitory action of organic acids is due to their ability to passively diffuse through the plasma membrane of the target cell and disrupt and/or inhibit certain metabolic activities. Among these acids, lactic acid is one of the most effective because it lowers the pH, which inhibits the growth of various microorganisms such as aflatoxigenic molds. As for bacteriocins, they have bactericidal and antifungal properties that may or may not be followed by cell lysis (Boudouma et al. 2023; Price‐Christenson and Yannarell 2023). Furthermore, the antiaflatoxigenic activity of lactic acid bacteria can also manifest itself through two main mechanisms of action: either by bioabsorption, which consists of absorbing the toxin in order to eliminate it, or by biodegradation by producing enzymes such as proteases and lactonases (Okeke et al. 2018; Okenge and Bombali 2025), which have the property of degrading the toxin. Indeed, these detoxification mechanisms are linked to a physical union between the toxin and the microorganism, and an adhesion to the components of the bacterial cell wall, in particular to polysaccharides and peptidoglycans (Nahle et al. 2022). This study is an important step in the validation process of *Lactobacillus* biopesticides formulation. The preliminary results are interesting, and it is planned to apply the formulations to a larger sample and to consider their application in a real environment in stores and fields.

## 4 Conclusion

The various extracts made with *Lactobacillus* isolates further highlight the possibilities offered by the knowledge and control of the antifungal and antiaflatoxigenic activities of this genus of bacteria in the field of biopreservation of foodstuffs in general and in particular cereals of major animal and human consumption.

For innovation in post‐harvest management of maize and the advancement of biopreservation methods, *Lactobacillu*s‐based preservation extracts with strong antifungal and antiaflatoxigenic activities were made from l *gapal* and local *fura*. Efficacy tests of these antifungal substances have prevented the development of molds that infect maize, especially aflatoxin‐producing strains, and have also degraded aflatoxins in infected maize.

It should be noted, however, that efforts to improve these extracts and their application procedures will be of much greater benefit if they are widely disseminated to all stakeholders in the maize industry. It would be possible to apply these formulations upstream in order to test the potential for protecting cereals in the field, such as Aflasafe.

## Author Contributions

**Inoussa Ilboudo:** conceptualization (equal), funding acquisition (equal), investigation (equal), methodology (equal), writing – original draft (equal), writing – review and editing (equal). **Hamidou Compaoré:** conceptualization (equal), methodology (equal), supervision (equal). **Inoussa Compaoré:** methodology (equal), supervision (equal). **Sheryl Mounira Traoré:** formal analysis (equal), investigation (equal), methodology (equal). **Laeticia Ella Dembélé:** formal analysis (equal), writing – original draft (equal). **Fulbert Nikièma:** resources (equal). **Hagrétou Sawadogo‐Lingani:** supervision (equal). **Lanterbecq Déborah:** resources (equal). **Elie Kabré:** resources (equal).

## Conflicts of Interest

The authors declare no conflicts of interest.

## Data Availability

The data used to support the findings of this study are available upon request from the corresponding author.
